# Establishment and characterization of a novel human induced pluripotent stem cell line stably expressing the iRFP720 reporter

**DOI:** 10.1038/s41598-022-12956-1

**Published:** 2022-06-14

**Authors:** Anita Fehér, Andrea Schnúr, Suchitra Muenthaisong, Tamás Bellák, Ferhan Ayaydin, György Várady, Elisabeth Kemter, Eckhard Wolf, András Dinnyés

**Affiliations:** 1 BioTalentum Ltd, Aulich Lajos Street 26, Gödöllő, 2100 Hungary; 2 Department of Anatomy, Histology and Embryology, Albert Szent-Györgyi Medical School, University of Szeged, Szeged, 6724 Hungary; 3 Functional Cell Biology and Immunology Advanced Core Facility, Hungarian Centre of Excellence for Molecular Medicine, University of Szeged (HCEMM-USZ), Szeged, 6720 Hungary; 4 Laboratory of Cellular Imaging, Biological Research Centre, Eötvös Loránd Research Network, Szeged, Hungary; 5 Research Centre for Natural Sciences, Institute of Enzymology, Budapest, 1117 Hungary; 6 Chair for Molecular Animal Breeding and Biotechnology, Gene Centre and Department of Veterinary Sciences, LMU Munich, 81377 Munich, Germany; 7 Centre for Innovative Medical Models (CiMM), Department of Veterinary Sciences, LMU Munich, 85764 Oberschleißheim, Germany; 8 German Center for Diabetes Research (DZD), 85764 Neuherberg, Germany; 9 HCEMM-USZ Stem Cell Research Group, Hungarian Centre of Excellence for Molecular Medicine, Szeged, 6723 Hungary; 10 Department of Cell Biology and Molecular Medicine, University of Szeged, Szeged, 6720 Hungary; 11 Department of Physiology and Animal Health, Institute of Physiology and Animal Nutrition, Hungarian University of Agriculture and Life Sciences, Gödöllő, 2100 Hungary

**Keywords:** Induced pluripotent stem cells, Stem cells, Stem-cell differentiation, Cellular imaging

## Abstract

Stem cell therapy has great potential for replacing beta-cell loss in diabetic patients. However, a key obstacle to cell therapy’s success is to preserve viability and function of the engrafted cells. While several strategies have been developed to improve engrafted beta-cell survival, tools to evaluate the efficacy within the body by imaging are limited. Traditional labeling tools, such as GFP-like fluorescent proteins, have limited penetration depths in vivo due to tissue scattering and absorption. To circumvent this limitation, a near-infrared fluorescent mutant version of the DrBphP bacteriophytochrome, iRFP720, has been developed for in vivo imaging and stem/progenitor cell tracking. Here, we present the generation and characterization of an iRFP720 expressing human induced pluripotent stem cell (iPSC) line, which can be used for real-time imaging in various biological applications. To generate the transgenic cells, the CRISPR/Cas9 technology was applied. A puromycin resistance gene was inserted into the *AAVS1* locus, driven by the endogenous *PPP1R12C* promoter, along with the CAG-iRFP720 reporter cassette, which was flanked by insulator elements. Proper integration of the transgene into the targeted genomic region was assessed by comprehensive genetic analysis, verifying precise genome editing. Stable expression of iRFP720 in the cells was confirmed and imaged by their near-infrared fluorescence. We demonstrated that the reporter iPSCs exhibit normal stem cell characteristics and can be efficiently differentiated towards the pancreatic lineage. As the genetically modified reporter cells show retained pluripotency and multilineage differentiation potential, they hold great potential as a cellular model in a variety of biological and pharmacological applications.

## Introduction

Over the past decade, cellular therapies have emerged as the new frontier for the treatment of various chronic diseases, including diabetes^1^. Because various types of diabetes are linked to a loss of beta cells, cell therapy research focuses on beta-cell replenishment strategies to compensate for insulin deficiency^2–4^. Human embryonic stem cells (hESC) and induced pluripotent stem cells (hiPSC) are considered as very attractive sources of surrogate beta cells, because of their ability to differentiate into all major somatic cell lineages, including endoderm, where the beta cells originate^5–7^. Pluripotent stem cells have the potential to address the shortage of cell source, and in addition to their renewable capacity, when patient-derived iPSCs are used the allogeneic immune response can also be avoided^8^.

Monitoring of cell homing and the fate of the delivered cellular products, including death, survival, proliferation, migration and differentiation, is fundamental for clarification of the regenerative process and its safety. Advanced biosafety visualization of the grafted cells and tracking their fate in the host has a great importance in preclinical assessment of novel cell-based therapies. This can be achieved by reporter gene expression or physical labelling using nanoparticles^9^. A reliable in vivo imaging method, in addition to monitoring cell viability, would also provide information on the in vivo migration of the transplanted cells, thus allowing a systematic investigation of cell therapy, which is crucial for proper scientific interpretation. Ideally, a bioimaging method should be the least invasive, non-toxic for the patient, and provide an exclusive visualization of the viable grafted cells.

Cell labelling with reporter genes such as green or red fluorescent proteins (eGFP, DsRed, mCherry) could provide an attractive option to trace transplanted cells^10–12^. The expression of these reporter genes generates easily measurable signal suitable for cell monitoring and changes in signal intensity can indicate cell death or proliferation. However, the use of this type of reporters is limited due to the low penetration depths of visible light and fading of the signal in the body^13,14^. The conventional GFP-reporter can be detected in the visible spectral range, where autofluorescence is relatively high, thereby significantly increasing the background and interfering with imaging^15^. Near-infrared fluorescent proteins (NIRFPs), developed from the DrBphP bacterial phytochrome of *Deinococcus radiodurans* overcome this limitation, because autofluorescence is generally much lower in the 700–1000 nm spectrum^16^. In addition, iRFP720 has been shown to be easily detected in vivo due to its minimal absorption in mammalian tissues, thus providing a new perspective in the application for protein labelling, live cell imaging and in vivo tracking^17^. This imaging approach offers the potential for earlier detection of rejection or dysfunction of the transplanted cells, tissues, or organs^18,19^. iRFP720 labelled cells have been efficiently used to mark ovarian cancer, lung cancer, and breast cancer as well as mesenchymal stem cell grafts in the brain^20–23^ and were able to track cell engraftment in heart^24–26^.

In this study, we established and characterized a novel human iPSC line that stably expresses the near-infrared fluorescent protein, by inserting the iRFP720 coding sequence into the *AAVS1* safe harbor locus of human iPSCs. To generate the genetically modified reporter iPSCs, the CRISPR/Cas9 technology was applied which is more efficient than the conventional methods and offers powerful tool for accurate genome editing to generate improved cellular models^27–29^. The newly established iRFP720 reporter hiPSC line might become an ideal tool for real-time imaging, enabling robust readout that helps both cell therapy development and drug screening applications.

## Results

### Targeting strategy

**Figure 1:**
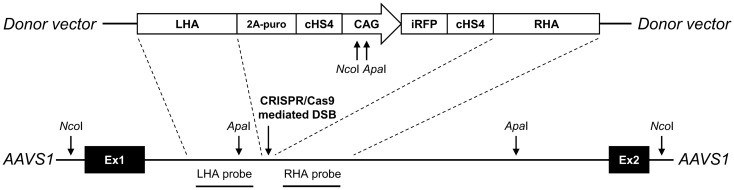
Targeting of the iRFP720 reporter construct into the *AAVS1* locus of SBAD2 hiPSCs. Donor vector used to target the locus is depicted above. LHA/RHA: Left and Right Homology Arms, 2A-puro: 2A self-cleaving peptide sequence and the puromycin resistance gene, cHS4: chicken hypersensitive site-4 insulator sequence, CAG: CMV early enhancer/chicken β actin promoter, iRFP: iRFP720 near-infrared fluorescent protein coding sequence. The first 2 exons of PPP1R12C gene in the *AAVS1* locus are shown with black boxes. CRISPR/Cas9 mediated DSB (double-strand break) and the restriction cut sites are indicated by arrows. The probes used for Southern blot analysis are depicted accordingly.

SBAD2 hiPSC line was used to generate the reporter cells by inserting a CAG-promoter driven iRFP720 cassette into the *AAVS1* safe harbor locus. CAG promoter was chosen after careful consideration of multiple promoter options. It enables strong and stable expression of transgenes/reporters with little risk of silencing and helps to detect the transplanted cells continuously, independently from their differentiation status and activation of tissue-specific promoters^30–32^. Instead of random insertion, the CRISPR/Cas9 technology was used for targeted integration of the reporter cassette into the *AAVS1* locus to prevent transgene silencing and position effect related expression variations that can affect the proper endogenous gene expression pattern. To generate the transgenic cell line, a puromycin resistance gene was inserted into the *AAVS1* locus, driven by the endogenous *PPP1R12C* promoter, along with the CAG-iRFP720 reporter cassette, which was flanked by cHS4 insulator elements to block the potential interactions between the transgene and the target cell genome (Fig. 1).

### Gene targeting, genetic screening, clone testing

**Figure 2:**
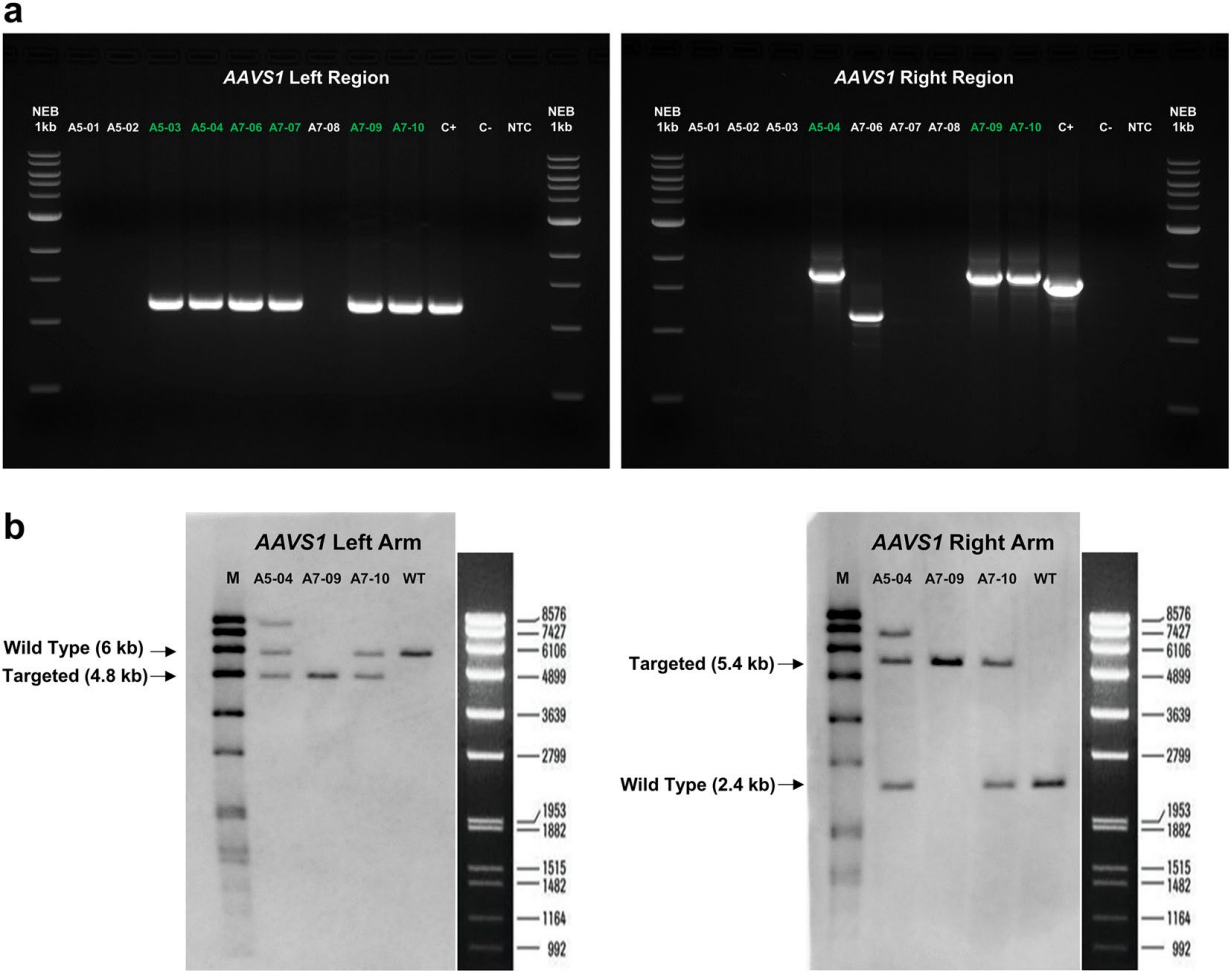
Genetic screening. (**a**) Junction PCRs were performed using locus-specific primers that bind to genomic sequences outside of the homology regions in combination with vector-specific primers. Expected fragment sizes for positive samples: 1196 bp for the left region and 1789 bp for the right region. Three clones tested positive in the screening that contained correctly integrated donor DNA at both ends. C+: positive control hiPSC line containing genome integrated eGFP sequence in the *AAVS1* locus, C-: negative control SBAD2 hiPSC line, NTC: no template control. The original uncropped gels are presented in Supplementary Figure S1. (**b**) Southern blot analysis of the candidate clones (A5-04, A7-09, A7-10) and negative control SBAD2 hiPSC line. gDNA samples were digested with *Nco*I or *Apa*I restriction enzymes and tested with *AAVS1*-LHA or *AAVS1*-RHA specific probes, respectively. WT: negative control SBAD2 hiPSC line, M: DIG-labeled DNA Molecular Weight Marker VII (Roche), the ladder scale specified by the manufacturer is shown on the right side of the blots. The original uncropped blots are presented in Supplementary Figure S2.

SBAD2 hiPSCs were nucleofected with the donor vector and CRISPR/Cas9 components (applied as RNP complexes), then the cells were selected by puromycin. Targeting efficiency was relatively low, with 0.001% of cells surviving the puromycin treatment. After selection, individual drug-resistant colonies were isolated, propagated, and screened by locus-specific junction PCRs (Fig. 2a, Supplementary Fig. S1). The junction PCR results showed that several clones contained only partially integrated donor sequences. Three correctly modified PCR-positive clones were found and then analyzed by Southern blot (A5-04, A7-09 and A7-10). The Southern blot experiments (Fig. 2b, Supplementary Fig. S2) showed proper integration of the reporter cassette into the *AAVS1* locus and confirmed the heterozygous targeting event in the A7-10 clone, demonstrating a single copy integration into the targeted genomic locus, while in case of A7-09 cells both *AAVS1* alleles were modified (biallelic targeting). An additional fragment was detected in A5-04 sample, suggesting extra vector-integration into another genomic location in this clone. Thus, the overall efficiency of precise genetic modification was found to be quite low (0.0002%), with a homozygote to heterozygote ratio of 1:2. DNA sequencing of the targeted genomic region in the A7-09 and A7-10 clones verified the accurate genome editing, and potential CRISPR/Cas9 mediated off-target cleavages were assessed by sequence analysis of the four most likely predicted off-target sites. The results showed that there was no CRISPR/Cas9 mediated nonspecific cleavage at any of these off-target sites.

### iRFP720 expressing hiPSCs show normal stem cell characteristics

**Figure 3:**
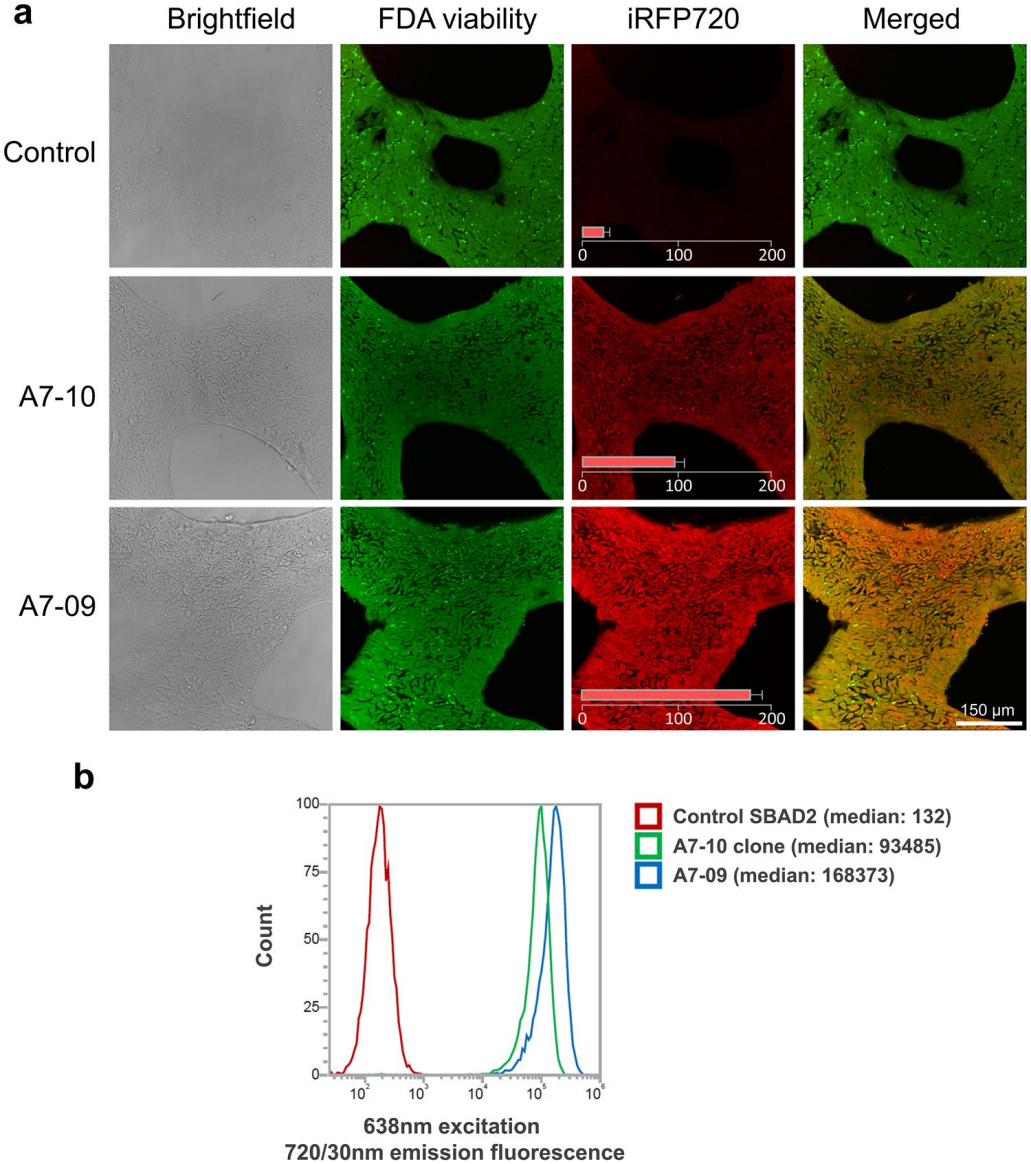
Live cell fluorescent imaging and FACS-analysis of the SBAD2-iRFP720 reporter hiPSCs. (**a**) iRFP720 expressing (red, excitation/detection: 633/LP650) and control cells were stained with viability indicator dye fluorescein diacetate (FDA, green, excitation/detection: 488/500–530). Bar charts on iRFP720 images show average fluorescence intensities (arbitrary intensity values) measured from 10 different 50 × 50 µm area. Scale bar: 150 µm. (**b**) Flow cytometry analysis of the iRFP720 expressing SBAD2 hiPSC clones. Excitation of 638 nm was used, and emission window was set to 720/30 nm. The clones were proved to be homogeneous for the reporter expression.

**Figure 4:**
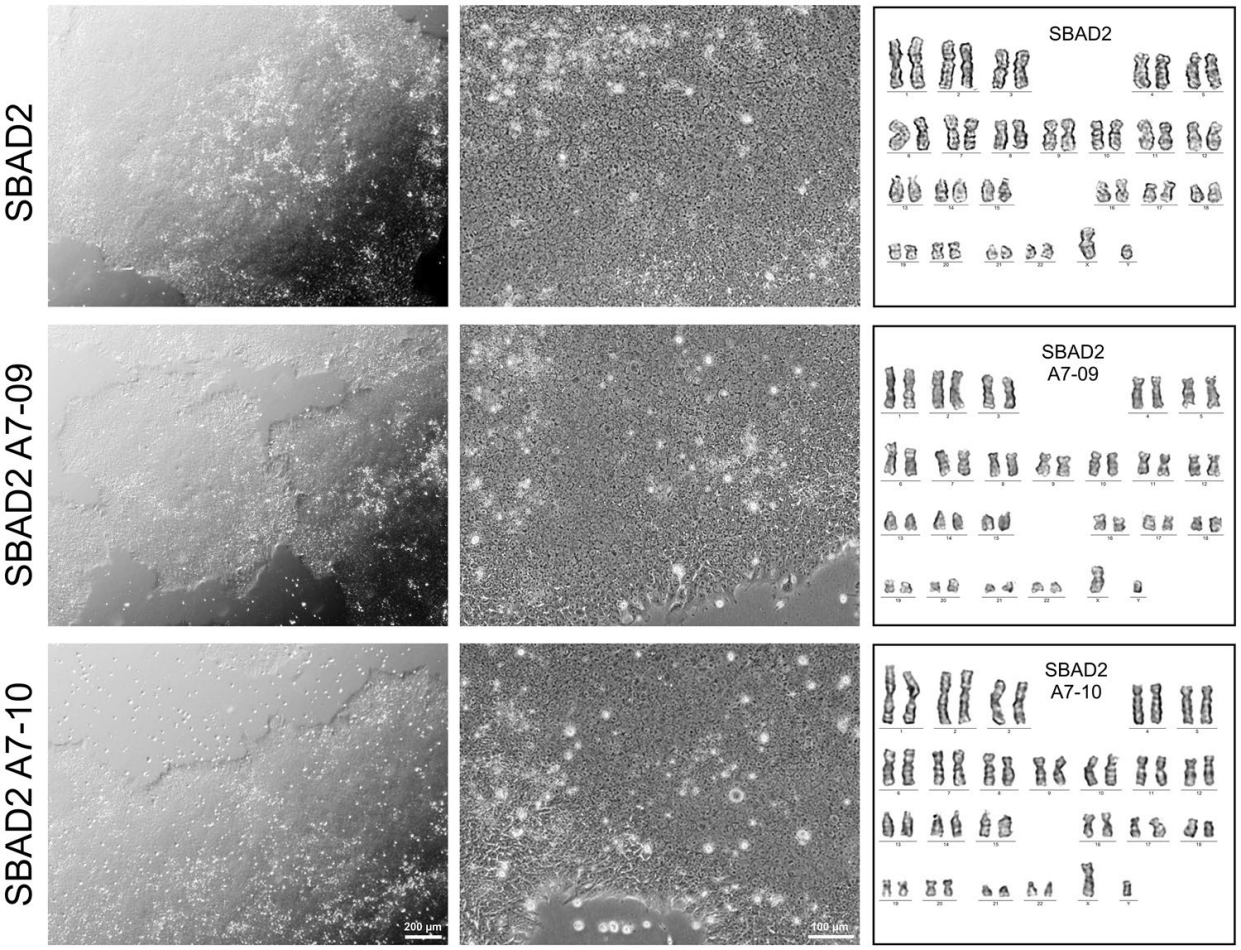
iRFP720 expressing hiPSCs show normal stem cell characteristics and karyotype. Brightfield images depicting the proper iPSC-morphology of the SBAD2 and SBAD2-iRFP720 reporter hiPSCs. Scale bars: 200 μm (left panels) and 100 μm (middle panels). Karyogram of the cell lines showed normal 46 chromosomes (XY).

**Figure 5:**
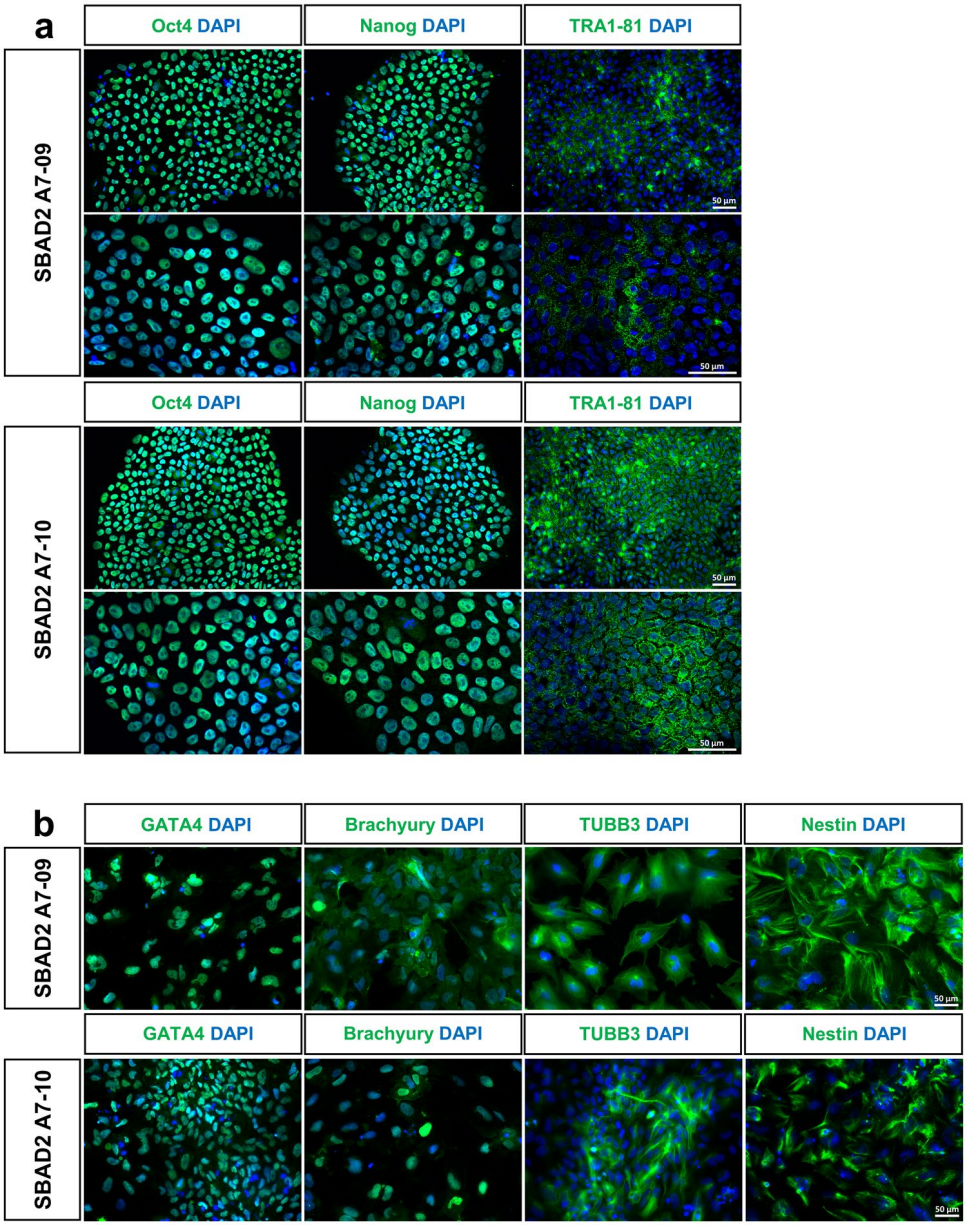
Pluripotency tests. (**a**) Representative immunofluorescent micrographs of undifferentiated SBAD2-iRFP720 reporter hiPSCs, that were positively stained for stem cell markers OCT4, NANOG and TRA1-81 (in green), nuclei were labeled with DAPI (in blue). (**b**) SBAD2-iRFP720 hiPSCs were spontaneously differentiated and analyzed by immunocytochemistry. Multilineage differentiation potential was confirmed by immunostaining for endodermal (GATA4), mesodermal (BRACHYURY) and ectodermal (TUBB3, NESTIN) germ layers (in green), nuclei were labeled with DAPI (in blue). Scale bar: 50 μm.

Two iRFP720 expressing hiPSC clones (A7-09 and A7-10) were further tested and characterized. Expression of the iRFP720 reporter gene was both detected with fluorescence live cell imaging (Fig. 3a) as well as by flow cytometry, confirming high-level of iRFP720 expression in both clones (Fig. 3b). iRFP720 fluorescence signal was 500–900-fold higher than the basal autofluorescence level and the cell populations were found to be homogeneous for the reporter expression. Based on microscopic fluorescence intensity quantification and flow cytometric measurements, A7-09 cells displayed 1.8 × more red emission as compared to A7-10 cells. Both clones showed proper hiPSC-morphology, the colonies were tightly packed, round-shaped, and the cells had a high nuclear/cytoplasm ratio, which was indistinguishable from that of the parental SBAD2 hiPSCs (Fig. 4). The iRFP720 reporter cell lines were also checked for chromosome-integrity and showed normal diploid 46, XY karyotype (Fig. 4). An important consideration when manipulating hiPSCs is the maintenance of pluripotency. Gene targeting is stressful, and it may have negative effect on cell survival and proliferative capacity. To confirm the pluripotency and multilineage differentiation ability of the reporter cells, embryoid bodies (EBs) were formed and cultured for 14 days in differentiation medium, then the differentiated cultures were characterized for the expression of the three germ layer markers. We found that the undifferentiated reporter iPSCs were positively stained for the major pluripotency markers (OCT3/4, NANOG, TRA-1-81, Fig. 5a) and they were able to differentiate into endodermal (GATA4), mesodermal (BRACHYURY) and ectodermal (TUBB3, NESTIN) lineages (Fig. 5b), confirming retained pluripotent stem cell properties of the iRFP720-expressing reporter cells.

### A7-10 iRFP720 expressing iPSCs are able to efficiently differentiate into pancreatic precursor cells

**Figure 6:**
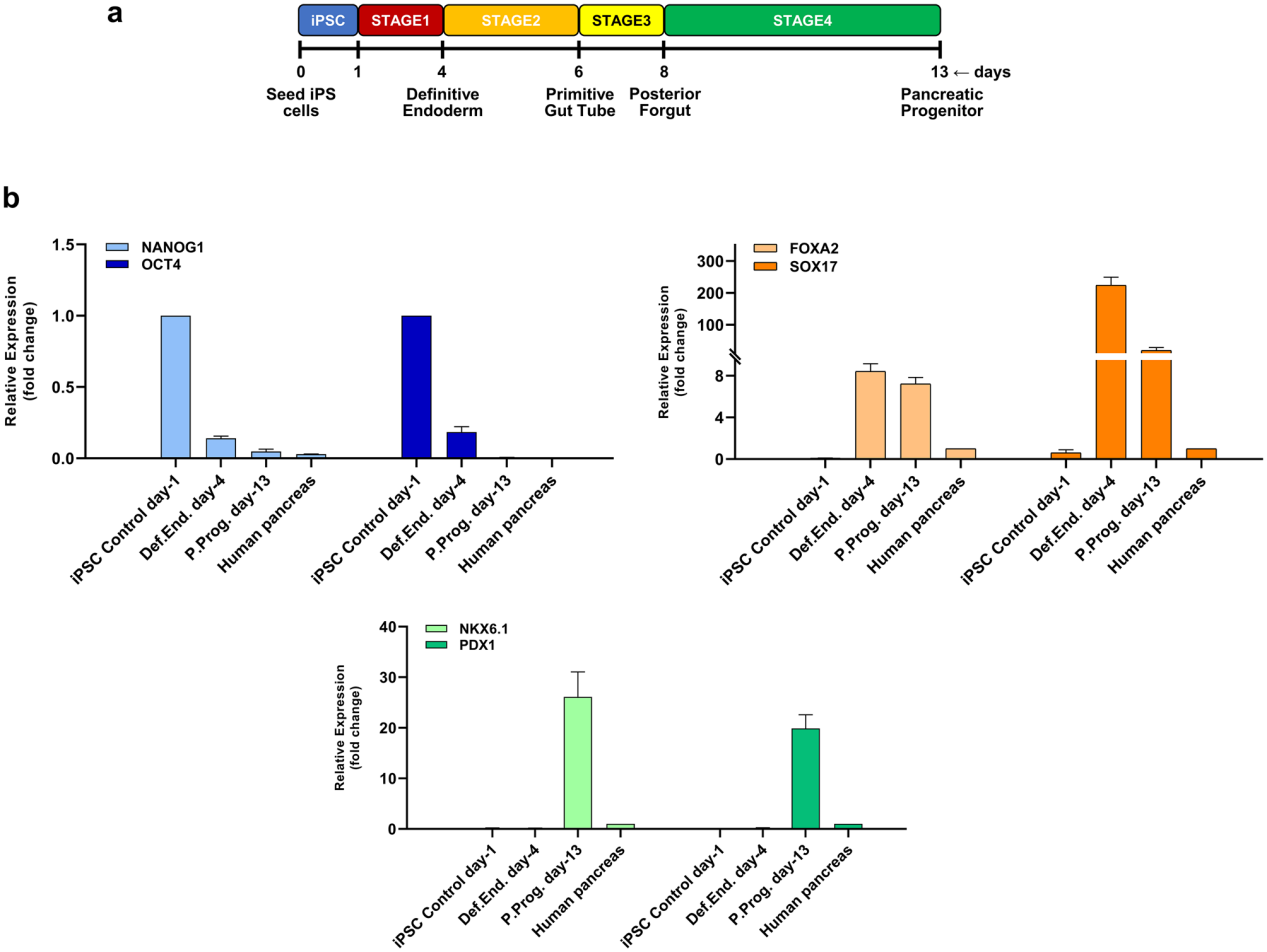
Pancreatic differentiation of SBAD2-iRFP720 reporter hiPSCs in 3D culture system. (**a**) Schematic for the generation of pancreatic progenitor cells. (**b**) mRNA expression of pluripotency markers (OCT4 and NANOG), definitive endoderm (FOXA2, SOX17) and pancreatic progenitor (NKX6.1, PDX1) markers at the indicated differentiation stages determined by RT-qPCR measurements. Data are presented as mean ± SEM, n = 3.

**Figure 7:**
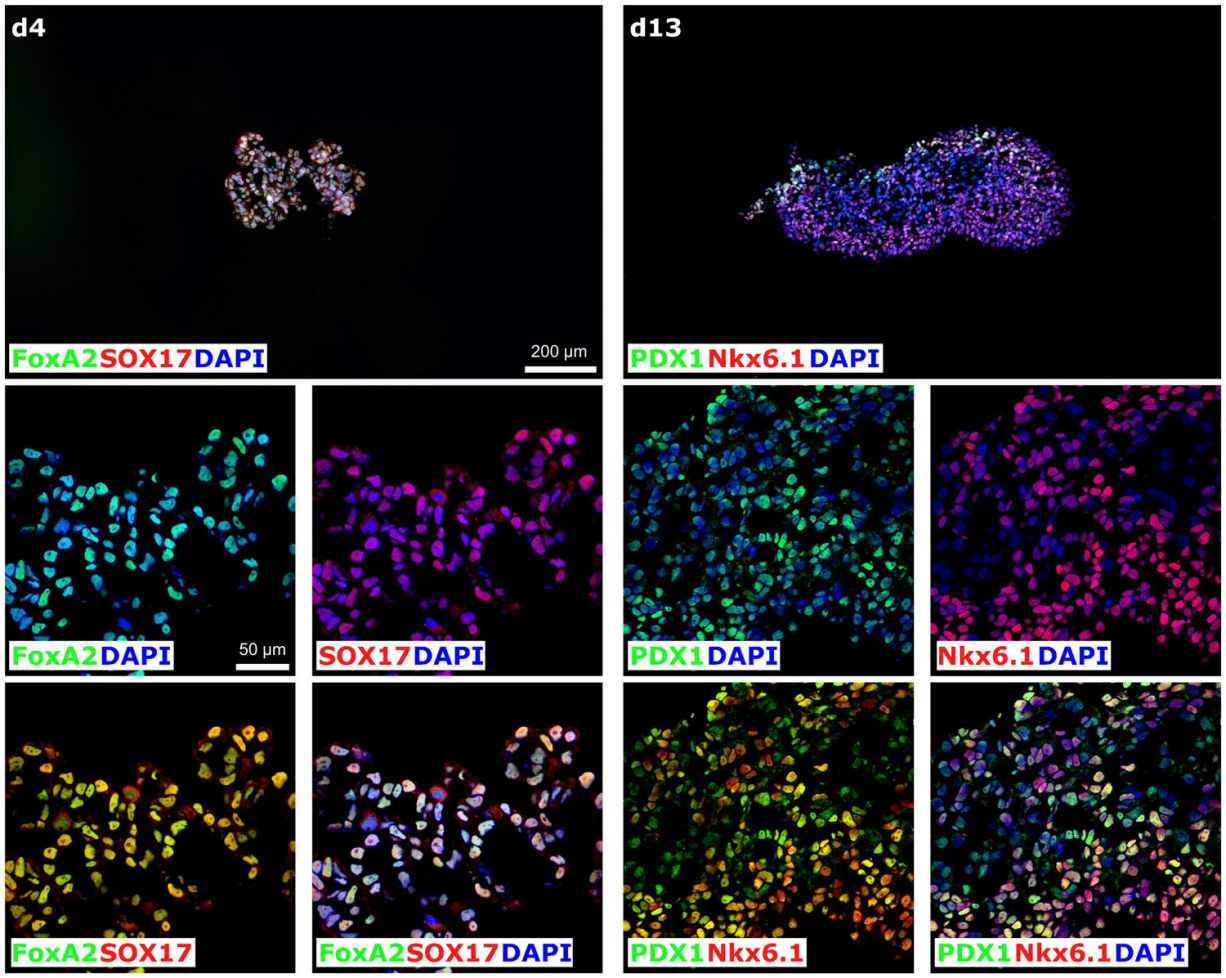
Immunocytochemical analysis of stage specific marker expression during pancreatic progenitor differentiation. SBAD2-iRFP720 reporter hiPSCs were differentiated in 3D culture system. Spheroids were fixed, cryosectioned, and the highest diameter middle sections were immunostained after 4 and 13 days of differentiation. Top panels represent the overview of the cryosectioned cultures, while the rest of the panels show higher magnifications on day 4 (left) or day 13 (right). On day 4, most of the cells express definitive endoderm markers FOXA2 (green) and SOX17 (red). Note the high expression of the key transcription factors in pancreatic cell development on day 13 (PDX1 in green, NKX6.1 in red). Nuclei were counterstained with DAPI (blue). Scale bars: 200 μm and 50 μm.

As summarized above, both clones were found to show well-detectable, homogeneous iRFP720 expression. Taking into account the potential induction of endoplasmic reticulum stress in the cells due to overexpression of the reporter protein, the heterozygous A7-10 clone was selected for further studies and to generate pancreatic progenitor cells expressing the iRFP720 reporter gene. We used three-dimensional (3D) culture system, as it has been reported that the induction rate of PDX1 + /NKX6.1 + pancreatic cells is markedly improved with cell aggregation, and this positive effect on pancreatic differentiation has been reproduced with multiple hESC/iPSC lines that were able to differentiate efficiently in 3D cultures^33^. Further advantages of these 3D aggregation cultures are that large-scale production is more easily achievable and the produced spheroids are readily accessible for transplantation in comparison to two-dimensional (2D) adherent cultures. Therefore, we tested pancreatic progenitor differentiation in a 3D culture system and found that the iRFP720 reporter cell line can be efficiently differentiated towards pancreatic lineage using well-established published protocols^67^. Cells were differentiated over a 13-days period by sequential media changes in a four-stage differentiation scheme (Fig. 6a). Samples were collected at definitive endodermal and pancreatic progenitor stages for assessment of stage specific differentiation markers by RT-qPCR and immunohistochemistry. The spheroids showed an appropriate marker expression profile at each stage, as genes and transcription factors related to pancreatic development were selectively upregulated at the specific stages: SOX17 and FOXA2 in definitive endoderm stage, PDX1 and NKX6.1 in pancreatic progenitors. In parallel, pluripotency marker expression (NANOG1, OCT4) downregulated upon differentiation (Fig. 6b). Immunohistochemical analysis also confirmed the proper expression of the stage-specific differentiation markers (Fig. 7).

### iRFP720 expression is stable during pancreatic differentiation

**Figure 8:**
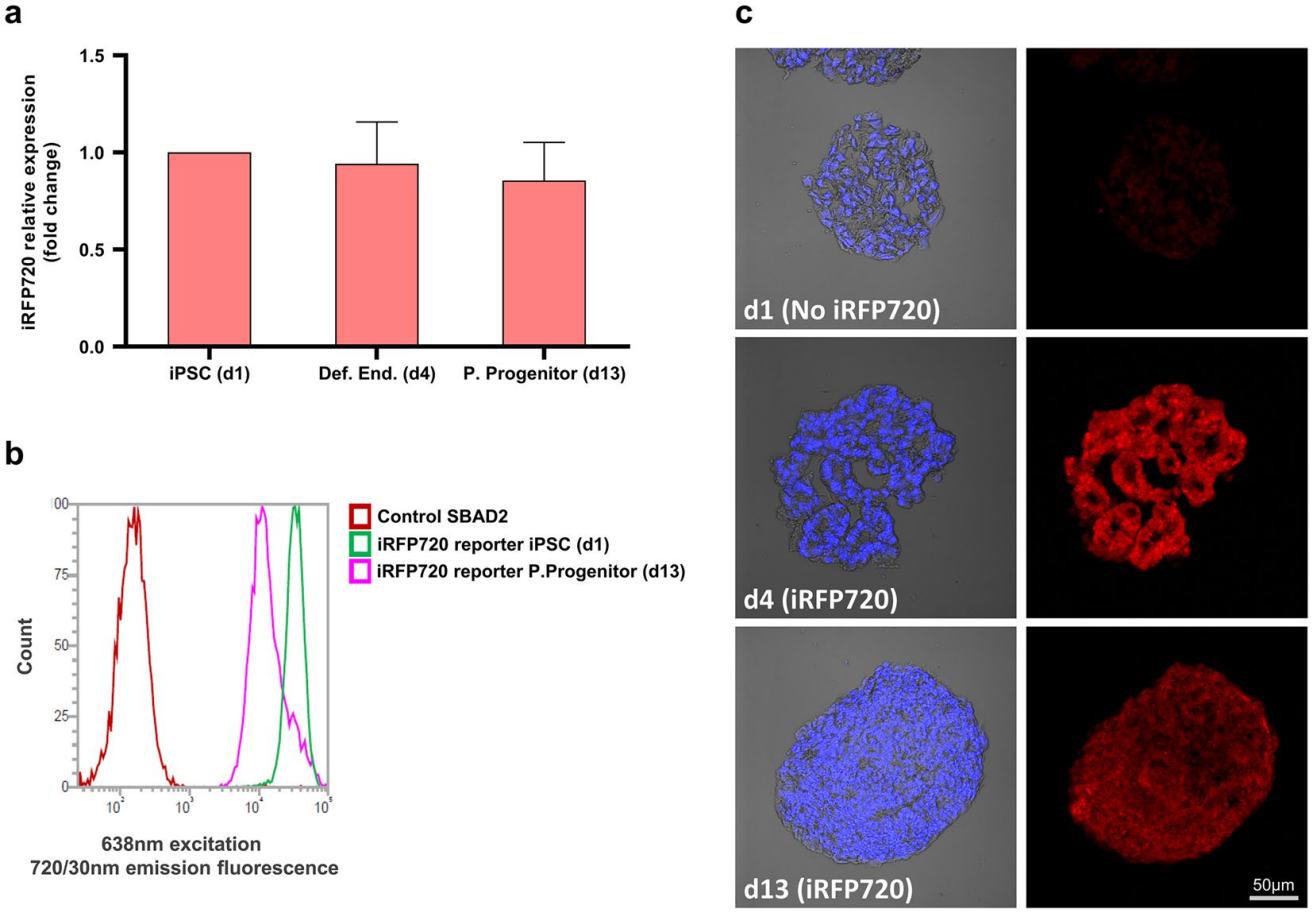
iRFP720 expression in SBAD2-iRFP720 reporter hiPSC-derived spheroids during pancreatic differentiation. Cells were differentiated for 13 days and analyzed. (**a**) iRFP720-mRNA expression at the indicated differentiation stages was determined by RT-qPCR measurements. Data are presented as mean ± SEM, n = 3. (**b**) iRFP720 fluorescence of control and reporter hiPSC-derived differentiated samples was measured by flow cytometry. Excitation of 638 nm was used, and emission window was set to 720/30 nm. (**c**) Confocal microscopic analysis of control (top panels) and iRFP720-expressing spheroids on day 4 and day 13 of pancreatic differentiation (middle and bottom panels). Left panels show representative merged images of the nuclear DAPI stain (blue) with bright field pictures of the cryosectioned spheroids, right panels demonstrate the iRFP720 expression (in red, excitation/detection: 633/LP650). Scale bar: 50 μm.

During hiPSC differentiation, epigenetic silencing by DNA methylation and/or loss of the transgene can contribute to reductions in the transgene expression^34,35^. Together, they present a major challenge in maintaining predictable and high yields of reporter protein expression. Therefore, we tested the stability of iRFP720 reporter expression during pancreatic differentiation. A7-10 reporter hiPSCs were differentiated into pancreatic progenitor cells with a 13-days differentiation protocol, and the cells were analyzed by RT-qPCR measurements, examined by flow cytometry, and imaged at various stages. We found no significant differences in iRFP720 mRNA expression during differentiation compared to the iPSC control (Fig. 8a). Although flow cytometry data and confocal microscopic analysis indicated slightly decreased fluorescent signal-intensity in pancreatic progenitor cells, we were still able to detect a strong iRFP720 transgene expression and fluorescence uniformly distributed in the differentiated spheroids (Fig. 8b-c).

## Discussion

Stem cell therapies provide unique opportunities for treating diabetes by replacing beta cells. The use of cadaveric donors to provide pancreatic Langerhans islets is impeded by a major lack of donor islets for transplantation. Regenerative medicine approaches using pluripotent stem cell derivates could provide a promising alternative source for replacing Langerhans islets^36^. Successful application of regenerative cell therapies requires a better understanding of cell fate after transplantation. This can be achieved by using molecular imaging that enables the longitudinal, non-invasive assessment of cellular behaviour in vivo after cell transplantation^37^. Cell tracking can be performed by labelling cells with molecular probes that enter the cell by active/passive transport and trapped intracellularly (direct labelling), or alternatively, by overexpression of specific reporter genes that integrate into the cellular genome (reporter gene labelling)^38^. Near-IR-based fluorescence imaging using iRFP720-labelling is a novel technology with potential use for in vivo applications^39^. However, at present, there are only a few studies on iRFP720-labeled stem cell tracking. The main goal of this study was to develop a genetically encoded iRFP720 reporter hiPSC line for multimodal optical imaging that would allow for continuous longitudinal non-invasive monitoring of transplanted stem cells and their derivatives with high sensitivity.

Several reporter cell lines have already been generated by inserting different promoter-driven reporter genes into the genome, e.g. into landing pads^40^, into the *CLYBL* locus on human chromosome 13^41–43^, or into the *AAVS1* locus^30,31,44,45^. The Adeno-Associated Virus Site 1 (*AAVS1*), located between exon-1 and intron-1 of *PPP1R12C* (protein phosphatase 1 regulatory subunit 12C) on human chromosome 19, considered favourable and well-characterized candidate in human iPSC transgenesis^46–50^. Insertion of exogenous DNA into the *AAVS1* locus promises predictable and strong transgene expression without noticeable functional and phenotypic alteration in the modified cell lines^46,51^. Based on these characteristics, *AAVS1* is commonly referred and used as a “safe-harbour” site. The expression of the transgene inserted into the *AAVS1* locus is robust and persistent, which on one hand explained by the maintenance of an open-chromatin configuration in this locus^52^. On the other hand, the presence of an insulator site has been found to prevent the spread of heterochromatin, thus ensuring stable transgene expression^51^. However, promoter silencing and clone-dependent variations in transgene expression has been observed in different cell types during directed differentiation when *AAVS1* site was used for transgene insertion, drawing attention to the need for careful clone-screening before and throughout the differentiation^53–56^.

In the presented study we targeted the iRFP720 reporter cassette flanked by additional insulator sequences into the *AAVS1* locus to prevent potential interactions between the transgene and the target cell genome. Protein-based CRISPR/Cas9 delivery system was used, which is known to be effective, immediate, and transient, thus less harmful to the cells than vector-based approaches. Due to a relatively short exposure time to the delivered CRISPR/Cas9-components, there is a lower risk for off-target events^57–59^. The frequency of HDR (Homology Directed Repair) which is needed for efficient incorporation of exogenous DNA sequences into the target locus is cell type-dependent, and in hiPSCs extremely low^60,61^. Due to the low HDR-efficiency in hiPSCs, the insertion of relatively long DNA like the coding sequence of fluorescent reporter cassettes is still difficult, labour-intensive and challenging, as demonstrated in the present study. Gene targeting results in a mixed cell population, therefore a refined selection process was needed to enrich for the successfully edited cells and to create homogeneous cell lines through subcloning, which was achieved by using puromycin selection. After verifying the correct genetic modification in the established reporter iPSC clones, they were subjected to detailed characterization. We have demonstrated the retained genomic stability, pluripotency and multilineage differentiation ability of the newly generated iPSC lines. Moreover, we have found that the reporter iPSCs were able to efficiently differentiate into pancreatic progenitor cells with maintained, uniform, and high level iRFP720 expression during the differentiation. Although a slight decrease was observed in the fluorescence intensity, the iRFP720 mRNA expression remained stable, indicating that there was no downregulation or transgene silencing, and this observation is more likely due to the metabolic shift which is a hallmark of differentiated cells^62–64^. Transgene expression in other lineages and specific cell types requires further investigation.

Overall, we anticipate that the newly developed iRFP720-reporter human iPSC line will become a valuable tool in a variety of biological applications: for real-time imaging and tracking of transplanted cells in preclinical studies to test novel cellular products in regenerative therapies as well as for applications in disease modelling, drug development and toxicological studies.

## Materials and methods

### Cell lines and in vitro cell culture conditions

In this study, SBAD202-01 human iPSC line (http://stembancc.org, ^65^), referred to as SBAD2 was used, which has been established from Normal Adult Human Dermal Fibroblast cells (Lonza, CC-2511) by reprogramming with non-integrative Sendai virus transduction. hiPSCs were cultured at 37 °C in a humidified atmosphere containing 5% CO_2_ in a feeder-free system on Matrigel (BD Biosciences)-coated tissue culture plates. Cells were maintained in mTeSR-1 culture medium (StemCell Technologies) which was changed daily, and the cells were passaged every 5–7 days using EDTA (0.02%, Versene, Lonza), according to the manufacturer's instructions. hiPSCs underwent routine mycoplasma screening and karyotyping.

### Donor vector construction

Expression cassette of iRFP720 under the control of a CAG promoter was obtained from pCAG-iRFP720 plasmid (Addgene_89687) and inserted into an *AAVS1* targeting vector backbone (Addgene_22212) through pCR-II-Blunt-TOPO cloning (Thermo Fisher Scientific). cHS4 insulator sequences, flanking the expression cassette and obtained from pLNHX_cHS4_650 plasmid, were also incorporated into the donor vector to protect reporter expression from silencing^66^.

### Transfection of donor vector and CRISPR/Cas9 elements

SBAD2 hiPSC culture at 70–80% confluency was incubated with Accutase (Sigma-Aldrich) at 37 °C for 9 min to prepare single-cell suspension for gene targeting. 8 × 10^5^ cells were nucleofected (in duplicates) with 22.5 µg CRISPR/Cas9 ribonucleoprotein (RNP) complex composed of GeneArt™ Platinum™ Cas9 protein (Thermo Fisher Scientific) and guide RNA (Supplementary Table S1), along with 3.5 µg donor vector, using Human Stem Cell Nucleofector Kit 1 (Lonza) and program B-016 in AMAXA Nucleofector™ 2b Device (Lonza). After nucleofection, the transfected cells were spread in 6 well plate and 1X RevitaCell Supplement (Thermo Fisher Scientific) was added into the mTeSR-1 culture medium to increase cell recovery. Puromycin selection started 2 days later with 800 ng/mL puromycin (Thermo Fisher Scientific) for 24 h then continued with 200 ng/mL concentration for a week. After selection, puromycin-resistant colonies were isolated, propagated and harvested for cryobanking and genetic analysis.

### Genetic screening

Junction PCRs were performed using locus-specific genomic primers that bind outside of the *AAVS1* homology regions, in combination with donor vector-specific primers (Supplementary Table S1). The fragments were amplified by Phusion Hot Start II High-Fidelity DNA Polymerase (Thermo Fisher Scientific). For Southern blot analysis, 5 μg genomic DNA was digested overnight with *Apa*I or *Nco*I restriction enzyme (NEB) and separated on agarose gel, then transferred onto Hybond N + nylon membrane (Amersham). DIG-labeled DNA probes were prepared by random primed labeling for the *AAVS1* left and right homology regions (581 and 622 bp, respectively). Probe labeling, hybridization and detection were performed using DIG-High Prime DNA Labeling and Detection Starter Kit II (Roche), following instructions of the manufacturer. Kodak Gel Logic 1500 Imaging System was used to document the gels and blots.

### Off-target analysis

The most likely off-target sites were predicted by the CRISPR design tool (http://CRISPR.mit.edu/) and the corresponding genomic regions were PCR-amplified using Phusion Hot Start II High-Fidelity DNA Polymerase. The PCR products were purified with GenElute PCR cleanup kit (Sigma-Aldrich) and sequenced directly using an ABI Prism 3130xl Genetic Analyzer and BigDye Terminator Cycle Sequencing v3.1 Kit (Applied Biosystems).

### Karyotyping

The iRFP720 reporter hiPSCs were treated with Demecolcine solution (10 μg/mL in Hanks' Balanced Salt solution (HBSS)) and processed with standard methods. Giemsa-banded karyotype analysis was performed for a minimum of 20 metaphase cells and the chromosomes were classified according to the International System of Human Cytogenetic Nomenclature (ISCN).

### Pluripotency tests

Cell clumps were cultured in suspension for five days in mTeSR-1. The formed embryoid bodies (EBs) were plated on 0.1% gelatin (Merck) coated surface in differentiation medium (DMEM, 20% FBS, 1% MEM Non-Essential Amino Acid Solution (100×), 0.1 mM β-mercaptoethanol, 1% Pen/Strep). On day 14 of differentiation, the cells were fixed with 4% formaldehyde solution and evaluated for the 3 germ layer markers by immunocytochemistry (Supplementary Table S2). Cells were analysed under a fluorescence microscope equipped with a 3D imaging module (Axio Imager system with ApoTome; Zeiss) controlled using AxioVision 4.8.1 software (Zeiss).

### Human induced pluripotent stem cell-derived pancreatic differentiation

For initiation of the differentiation process and to form three-dimensional spheroids, SBAD2-A7-10 iPSCs were seeded in 6-well low attachment plates (Costar, 3471) at 9 × 10^5^ cells/mL density in mTeSR-1 media supplemented with RevitaCell. The cells were then placed on an orbital shaker (MaxQ 2000 CO_2_) for overnight, set at rotation rate 95 rpm in a 37 °C incubator, 5% CO_2_, and 100% humidity. The differentiation was started next day by changing mTeSR-1 to stage specific differentiation medium. During the procedure, media changes were performed according to previously published protocol^67^.

### Flow cytometry

iPSC cultures and differentiated spheroids were dispersed into single-cell suspension by Accutase and Trypsin, respectively, then the cells were suspended in PBS containing 10 mM HEPES and analyzed using an Attune NxT flow cytometer (Thermo Fisher Scientific). iRFP720 fluorescence was measured with 638 nm excitation and emission at 720/30 nm. Analysis of the results was performed using Attune NxT 3.1.2 software.

### Live cell iRFP imaging

Live cells were washed once with PBS and incubated at room temperature for least 5 min in 5 µg/mL fluorescein diacetate (FDA) working solution which was prepared freshly in PBS from a thawed stock solution of 5 mg/ml FDA in DMSO. Olympus FV1000 confocal laser scanning microscope was used for imaging of cells with 488 nm excitation and 500–530 nm emission ("fluorescein" dye combination) for FDA, or with 633 nm excitation and LP650 nm emission ("Cy5" dye configuration) for iRFP720.

### Immunocytochemistry of three-dimensional cultures

Cryosectioning and immunocytochemistry were performed based on our previously described methods^68^. 3D spheroid samples were fixed with 4% formaldehyde in 0.1 mol/L PBS for 1 h at room temperature (RT) and washed 3 times with PBS. The fixed cultures were then cryoprotected in 30% sucrose in PBS containing 0.01% sodium-azide at 4 °C until embedding in Shandon Cryomatrix gel (Thermo Fischer Scientific). 16-μm parallel sections were prepared using cryostat (Leica CM 1850 Cryostat, Leica GmbH), mounted to Superfrost™ Ultra Plus Adhesion Slides (Thermo Fisher Scientific) and stored at -20 °C until use. After 10 min air-drying, the sections were permeabilized with 0.1% Triton X-100 in PBS and blocked for 1 h at 24 °C with 3% BSA in PBS. The sections were then incubated with primary antibodies (overnight, 4 °C; Supplementary Table S2). On the next day, the sections were washed 3 times in PBS, the isotype specific secondary antibodies were diluted in blocking buffer and applied for 1 h at RT. The sections were then washed again 3 times with PBS and covered using Vectashield® mounting medium containing DAPI (1.5 μg/mL; Vector Laboratories) (1 h, RT). Negative controls for the secondary antibodies were prepared in the same way by omitting the primary antibodies.

Highest diameter middle sections with immunoreactivity were analyzed using a BX-41 epifluorescent microscope (Olympus) equipped with a DP-74 digital camera and its CellSens software (V1.18; Olympus,) or using an Olympus FV-10i-W compact confocal microscope system (Olympus) with Fluoview FV10i software (V2.1; Olympus). For iRFP720-imaging of cryosectioned and DAPI stained spheroids (Fig. 8c), Olympus FV1000 laser scanning confocal microscope was used with "DAPI" and "Cy5" (iRFP720) dye settings. All images were further processed using the GNU Image Manipulation Program (GIMP 2.10.0) and NIH ImageJ analysis software (imagej.nih.gov/ij).

### RT-qPCR

Total RNA was isolated from 25 to 35 spheroids per sample using RNAqueous Micro Total RNA isolation Kit (Thermo Fisher Scientific) according to the manufacturer's instructions. RNA was transcribed by Maxima First Strand cDNA synthesis kit with DNase (Thermo Fisher Scientific). The amplification reactions were carried out in a total volume of 15 μL with SYBR Green JumpStart Taq ReadyMix (Sigma-Aldrich). Human *18S* rRNA was used as a reference. The data were analyzed by REST software (2009 V2.0.13), and the values are expressed as mean ± SEM. Oligonucleotide primers used in this study are listed in Supplementary Table S1.

## Supplementary Information

Supplementary Information.

## Data Availability

All data generated and analysed during this study are included in this published article and its Supplementary Information files. The vector sequence constructed in the current study has been uploaded to the GenBank repository and publicly available in https://www.ncbi.nlm.nih.gov/genbank/, under GenBank accession number: ON012604 (Targetting vector AAVS1 CAG iRFP720). iRFP720 protein sequence is available at the following link: https://www.fpbase.org/protein/irfp720. Images generated during this study have been deposited into the Cell Image Library and can be found at: http://cellimagelibrary.org/groups/54732. Targeting vector and the created cell lines are available from the corresponding author on request.
