# Supplementary material for: Establishment and characterization of a novel human induced pluripotent stem cell line stably expressing the iRFP720 reporter
Source: Sci Rep. 2022 Jun 14;12:9874. doi: 10.1038/s41598-022-12956-1 (PMC9198085; doi:10.1038/s41598-022-12956-1)
Supplement: Supplementary file 1 — Supplementary Information. [file 41598_2022_12956_MOESM1_ESM.pdf]

Supplementary Information

Establishment and Characterization of a Novel Human Induced Pluripotent Stem Cell Line Stably Expressing the iRFP720 Reporter

Anita Fehér[1#], Andrea Schnúr[1#], Suchitra Muenthaisong[1], Tamás Bellák[1,2], Ferhan Ayaydin[3,4], György Várady[5], Elisabeth Kemter[6,7,8], Eckhard Wolf[6,7,8], András Dinnyés[1,9,10, 11*]

[1] BioTalentum Ltd, H-2100, Gödöllő, Hungary

[2] Department of Anatomy, Histology and Embryology, Albert Szent-Györgyi Medical School, University of Szeged, H-6724, Szeged, Hungary

[3] Functional Cell Biology and Immunology Advanced Core Facility, Hungarian Centre of Excellence for Molecular Medicine, University of Szeged (HCEMM-USZ); H-6720 Szeged, Hungary

[4] Laboratory of Cellular Imaging, Biological Research Centre, Eötvös Loránd Research

Network, Szeged, Hungary

[5] Research Centre for Natural Sciences, Institute of Enzymology, H-1117, Budapest, Hungary

[6] Chair for Molecular Animal Breeding and Biotechnology, Gene Centre and Department of Veterinary Sciences, LMU Munich, 81377 Munich, Germany

[7] Centre for Innovative Medical Models (CiMM), Department of Veterinary Sciences, LMU Munich, 85764 Oberschleißheim, Germany

[8] German Center for Diabetes Research (DZD), 85764 Neuherberg, Germany

[9] HCEMM-USZ Stem Cell Research Group, Hungarian Centre of Excellence for Molecular Medicine, H-6723 Szeged, Hungary

[10] Department of Cell Biology and Molecular Medicine, University of Szeged, H-6720 Szeged, Hungary

[11] Department of Physiology and Animal Health, Institute of Physiology and Animal Nutrition, Hungarian University of Agriculture and Life Sciences, H-2100, Gödöllő, Hungary

[#]These authors contributed equally to this work.
*To whom correspondence should be addressed: András Dinnyés, BioTalentum Ltd, Aulich Lajos street 26. H-2100, Gödöllő, Hungary, Phone: +36 20 510 9632, email: andras.dinnyes@biotalentum.hu.

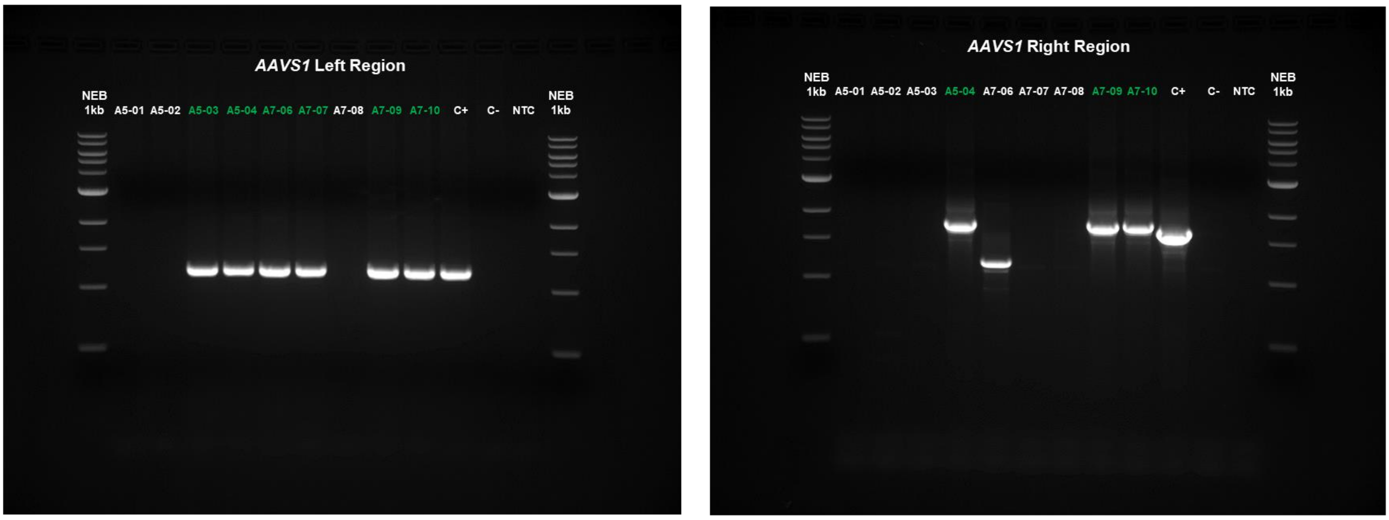

Supplementary Figure S1. Original uncropped gel pictures of the junction PCRs.

Junction PCRs were performed using locus-specific primers that bind to genomic sequences outside of the homology regions in combination with vector-specific primers. Expected fragment sizes for positive samples: 1196 bp for the left region and 1789 bp for the right region. Three clones tested positive in the screening that contained correctly integrated donor DNA at both ends. C+: positive control hiPSC line containing genome integrated eGFP sequence in the *AAVS1* locus, C-: negative control SBAD2 hiPSC line, NTC: no template control.

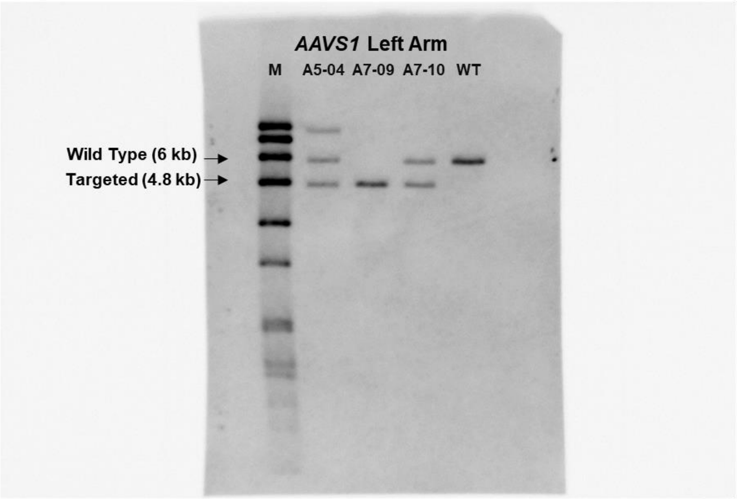

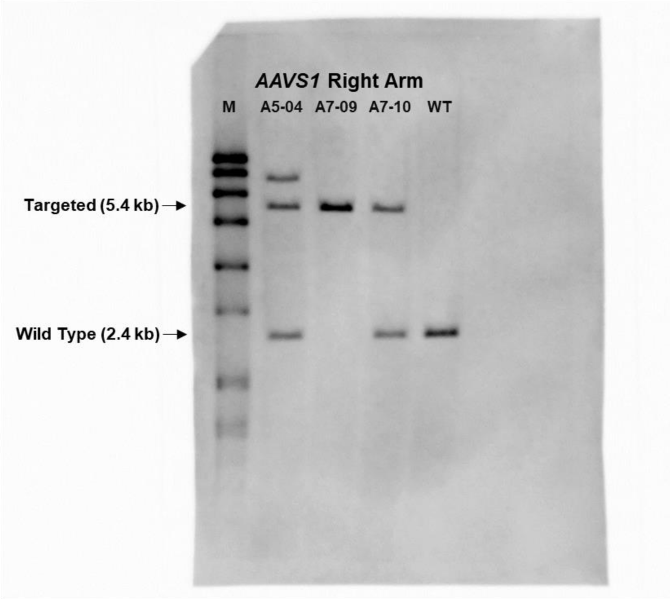

Supplementary Figure S2. Original uncropped Southern blot images.

Southern blot analysis of the candidate clones (A5-04, A7-09, A7-10) and negative control SBAD2 hiPSC line. gDNA samples were digested with *Nco*I or *Apa*I restriction enzymes and tested with *AAVS1*-LHA or *AAVS1*-RHA specific probes, respectively. WT: negative control SBAD2 hiPSC line, M: DIG-labeled DNA Molecular Weight Marker VII (Roche).

Name of the sequence/primers	Forward (5'-3')	Reverse (5'-3')	Application
**guide RNA**	ccactagggacaggattggtgac	gtcaccaatcctgtccctagtgg	Gene targeting
**Left-genomic**	cggaactctgccctctaacg		Screening
**Left-vector**		agctgcaagaactcttcctca	Screening
**Right-genomic**		tggaggagaatccacccaaa	Screening
**Right-vector**	cccagtcatagctgtccctct		Screening
**OFF-1**	cttggtggagagagagtgtgg	gcaccagaggctgatgactt	Off-target
**OFF-2**	gacattggccttgtggattt	tggattctgcccaagacact	Off-target
**OFF-3**	tgcagaatttgggatgagtg	cactcccttgctctggttct	Off-target
**OFF-4**	gtacagactggaggccagga	aggctgagtttatgggctga	Off-target
**OCT4**	gtggaggaagctgacaacaa	tcactcggttctcgatactgg	RT-qPCR
**NANOG1**	ctttgaagcatccgactgtaa	tttcttcaggcccacaaatc	RT-qPCR
**FOXA2**	agcggtgaagatggaaggg	cctcgggctctgcatagtag	RT-qPCR
**SOX17**	ggtgtgaatctccccgacag	taatataccgcggagctggc	RT-qPCR
**PDX1**	tgatactggattggcgttgt	taaacaggtcccaaggtgga	RT-qPCR
**NKX6.1**	ggcatcaacgatatcctgagc	gacgaggaagaggaggagga	RT-qPCR
**iRFP720**	caagaggtgcggaagattacc	ggatgaatgaggcaggatagtg	RT-qPCR
**18S**	gattaagtccctgccctttgta	agtcaagttcgaccgtcttctc	RT-qPCR

Supplementary Table S1. The guide RNA and PCR primer sequences used in the study.

	Antibody (used dilutions)	Company (Cat #)
**Primary Antibodies**	Mouse anti-Oct3/4 (1:50)	Santa Cruz Biotechnology Cat# sc-5279, RRID: AB_62805
	Goat anti-Nanog (1:100)	R&D Systems Cat# AF1997, RRID: AB_355097
	Mouse anti-TRA-1-81 (1:50)	Santa Cruz Biotechnology Cat# sc-21706, RRID: AB_628386
	Mouse anti-GATA4 (1:50)	Santa Cruz Biotechnology Cat# sc-25310, RRID: AB_627667
	Rabbit anti-Brachyury (1:50)	SantaCruz Biotechnology (sc-20109), RRID: AB_2255702
	Mouse anti-β3 Tubulin (1:50)	SantaCruz Biotechnology (sc-58888), RRID: AB_1119489
	Mouse anti-Nestin (1:1000)	Sigma Aldrich (MAB5326), RRID: AB_2251134
	Goat anti-FoxA2 (1:100)	R&D Systems (AF2400), RRID: AB_2845414
	Mouse anti-SOX17 (1:100)	Abcam (ab84990), RRID: AB_1861437
	Rabbit anti-PDX1 (1:500)	Abcam (ab47267), RRID: AB_777179
	Mouse anti-Nkx6.1 (1:100)	DSHB (F55A10), RRID: AB_532378
**Secondary Antibodies**	Alexa Fluor 488 donkey anti-mouse IgG (1:2000)	Thermo Fisher Scientific (A21202), RRID: AB_141607
	Alexa Fluor 594 donkey anti-mouse IgG (1:600)	Thermo Fisher Scientific (A21203), RRID: AB_141633
	Alexa Fluor 488 donkey anti-goat IgG (1:600, 1:2000)	Thermo Fisher Scientific (A11055), RRID: AB_2534102
	Alexa Fluor 488 goat anti-rabbit IgG (1:600)	Thermo Fisher Scientific (A11008), RRID: AB_143165

Supplementary Table S2. List of antibodies used in this study for ICC.
